# Circular RNA microarray expression profile and potential function of circ0005875 in clear cell renal cell carcinoma

**DOI:** 10.7150/jca.48770

**Published:** 2020-10-18

**Authors:** Qi Lv, Chunhui Ma, Haoming Li, Xuefeng Tan, Gangmin Wang, Yinan Zhang, Peijun Wang

**Affiliations:** 1 Department of Medical Imaging, Tongji Hospital, Tongji University School of Medicine, Xincun road No. 389, Shanghai, China; 2 Department of Orthopedics, Shanghai general hospital of Shanghai Jiaotong university, WujinRoad No. 85, 200080, shanghai, China.; 3 Department of Human Anatomy and Neurobiology, Nantong University, School of Medicine, Qixiu road No. 19, Nantong 226001, Jiangsu, China.; 4 Department of Urology, Huashan Hospital, Fudan University, Urumuqi Road No.12, 200040, Shanghai, China; 5 Department of Urology, Shandong Provincial Hospital Affiliated to Shandong First Medical University, Jingwuweiqi Road No.324, Jinan 250001, Shandong, China.

**Keywords:** circRNAs, clear cell renal cell carcinoma, microarray analysis, miRNA sponge, biomarker, hsa_circ_0005875

## Abstract

**Background:** Circular RNAs (circRNAs), a novel class of endogenous noncoding RNAs, are involved in a variety of diseases, including several types of cancers. We hypothesized that circRNAs are involved in the tumorigenesis and development of clear cell renal cell carcinoma (ccRCC).

**Methods**: To verify our hypothesis, we explored the circRNA expression profiles in 4 pairs of ccRCC tissues and their adjacent non-carcinoma tissues via microarray analysis. Selected circRNAs were further validated by qPCR. Moreover, hsa_circ_0005875 was selected for further study and the potential clinical values of hsa_circ_0005875 were investigated in 60 pairs of ccRCC tissues and adjacent normal controls. In addition, the role of hsa_circ_0005875 in ccRCC progression were performed using colony formation assay, Transwell assay and Martrigel-Transwell assay respectively. Finally, interactions between the circRNAs and miRNAs were predicted using Arraystar's miRNA target prediction software. Luciferase reporter assays were performed to evaluate the interaction between hsa_circ_0005875 and hsa_miR-145-5p.

**Results:** The microarray data showed 1988 circRNAs were significantly dysregulated circRNAs, including 1033 upregulated and 955 downregulated ones in the ccRCC tissues. Hsa_circ_0005875 was confirmed to be significantly upregulated in the ccRCC tumor tissues and renal carcinoma cells. Further analysis revealed that hsa_circ_0005875 expression was associated with tumor size, pathological TNM stage, histological differentiation, and lymphatic metastasis. Functional experiments demonstrated that overexpression of hsa_circ_0005875 increased proliferation, migration and invasion abilities. Moreover, bioinformatics analysis and luciferase reporter assays suggest that hsa_circ_0005875 may serve as a ceRNA (competing endogenous RNA) of miR-145-5p to relieve the repressive effect of miR-145-5p on target ZEB2.

**Conclusions:** These data indicate that hsa_circ_0005875 might play a role in promoting tumor growth and metastasis and be a potential biomarker of ccRCC.

## Introduction

Clear cell renal cell carcinoma (ccRCC), the most frequent and aggressive subtype of renal cell carcinoma (RCC), accounts for approximately 80% of all RCC cases. Although surgical excision is the most curative treatment for patients, nearly 30% patients develop metastasis after radical nephrectomy 1. Moreover, ccRCC patients generally respond poorly to radiotherapy and chemotherapy 2. Although targeted therapy has seen advances in recent years, the recurrence and metastasis in most patients are the main problems for ccRCC therapy. Given the unfavorable progress of ccRCC, it is important to establish a new reliable and more targeted strategy, which can contribute to the effectiveness of current cancer therapeutics.

Circular RNAs (CircRNAs) are a novel class of endogenous noncoding RNAs that are mainly formed by RNA splicing of the 5' end of the upstream exon and 3' end of the downstream exon 3,4. CircRNAs were previously regarded as byproducts of splicing mistakes or gene rearrangements, but recent accumulating evidence has shown that circRNAs may be crucial players in atherosclerosis 5, neurological disorders 6, diabetes 7, and a variety of human tumors 8-11. CircRNAs are one of the most promising new stars in the RNA family after microRNAs (miRNAs). Recently, it was confirmed that circRNAs can interact with miRNAs and function as sponges to arrest miRNAs from regulating gene expression through a circRNA-miRNA-mRNA pathway 12,13. As miRNA sponges, circRNAs competitively inhibit the transcriptional regulation of miRNAs. This unique characteristic of acting as a competing endogenous RNA (ceRNA) provides new ideas for drug development, and their tissue specificity and stability make circRNAs better biomarkers. However, there are no reports(there is no report) on circRNAs in ccRCC so far.

In this study, we hypothesized that circRNAs are involved in the tumorigenesis or development of ccRCC. To verify our hypothesis, we investigated circRNA expression profiles in ccRCC tissues via microarray analysis and validated our results in cancer tissues and their paired adjacent non-carcinoma tissues. Next, we analyzed the relationship between the aberrantly expressed circRNAs and the clinicopathological factors of patients with ccRCC. Our results provided a candidate diagnostic and prognostic biomarker for ccRCC and laid the foundation for further exploration of the mechanisms of circRNAs in ccRCC.

## Materials and methods

### Patients and tissue samples

Sixty four pairs of fresh-frozen samples with histologically confirmed ccRCC and matched para-carcinoma tissues were obtained from the Urology Department at Tongji Hospital (Shanghai, People's Republic of China) and Shandong Provincial Hospital Affiliated to Shandong First Medical University (Jinan, People's Republic of China) from June 2015 to July 2016.

Their diagnoses were independently rereviewed(reviewed) by two pathologists and classified by WHO criteria. The mean age of patients at diagnosis was 61.12±10.65 years (range, 38-80 years), with 34 males and 30 females. The inclusion criteria of the study subjects: 1 ccRCC was confirmed by pathological examination; 2 The selected patients had not received adjuvant treatment before radical resection; 3 The follow-up data were complete. Exclusion criteria: exclude other kinds of renal carcinoma such as chromophobe cell carcinomas and renal papillary carcinoma, etc. All patients were subject to clinical, imaging, and pathological diagnosis (**Supplementary Table 1**). Four paired samples from stage II (moderate differentiation, no lymphatic metastasis) and III (poor differentiation, lymphatic metastasis) balanced for gender were selected for microarray analysis of circRNAs (**Table 1**). All the tissue specimens were fresh-frozen in liquid nitrogen immediately after resection until further study. Clinical information was obtained from medical records at Tongji hospital and Shandong Provincial Hospital. The characteristics of the patients are summarized in Table 1. Informed consent was approved and reviewed by The Ethics Boards of Tongji Hospital and Shandong Provincial Hospital. All the experiments were carried out in accordance with institutional guidelines. Written informed consents were obtained from the patients undergoing surgery.

### RNA Extraction

Total RNA was extracted from the frozen renal tissues by using TRIzol reagent (Invitrogen, Carlsbad, CA, USA) according to the manufacturer's instructions. RNA quality and concentration were determined from OD260/280 readings using the NanoDrop ND-1000 spectrophotometer (NanoDrop Technologies, Montchanin, DE) and assessed via 1% gel electrophoresis, respectively.

### Microarray data analysis

The human CircRNA Array (Arraystar, USA) with each array containing probes interrogating about 5396 human circRNAs was used in this study. In brief, the total RNA sample was digested with Rnase R (Epicentre Inc.) to eliminate linear RNAs and enrich circular RNAs. Subsequently, each purified RNA sample was amplified and transcribed into cRNA by applying a random priming method (Arraystar Super RNA Labeling Kit; Arraystar). Next, the labeled cRNAs were hybridized onto the circRNAs and incubated for 17 h at 65°C in an Agilent Hybridization Oven. Finally, the arrays were washed and fixed, and the images were scanned using the Agilent Scanner G2505C (Agilent, Santa Clara, CA). Data analysis was performed using Agilent Feature Extraction software (version 11.0). Expression data were quantile normalized and processed using the R software limma package. The significantly differentially expressed circRNAs between the ccRCC group and the para-carcinoma group were screened based on fold change, *P*-value, and raw intensity. The required microarray information has been input into the Gene Expression Omnibus (serial number: GSE100186).

### Quantitative Real-Time PCR (qRT-PCR)

Quantitative real-time PCR was used to validate microarray data. Total extracted RNAs in tissue samples and relative cell lines including relative ccRCC cells and human renal proximal tubular epithelial cell line HK-2 as normal control were reverse transcribed using PrimeScript RT reagent Kit with gDNA Eraser (TaKaRa, Dalian, China) according to the manufacturer's protocol. Divergent primers of 20 circRNAs, including 10 upregulated and 10 downregulated ones, were screened to design primers, and β-actin was used as an internal control. The melting curve was drawn to ensure primer specificity. The expression of the circRNAs was calculated as mean ± standard deviations (SD) relative to the expression of the internal control gene, β-actin, to normalize the data.

### Annotation and Function Prediction for the circRNAs and bioinformatics analysis

Interactions between the circRNAs and miRNAs were predicted using Arraystar's miRNA target prediction software based on TargetScan and miRanda. Six validated circRNAs were annotated in detail using the circRNA/miRNA interaction information). Next, an mRNA-miRNA-circRNA network was established based on the consistent target miRNAs of the mRNAs and circRNAs. Pathway analysis and Gene Ontology (GO) were performed to identify significant molecular functions of the target miRNAs of the circRNAs.

### Cell culture and transfection

Human ccRCC cell lines caki-1,786-O,769-p, ACHN, A498 and HK-2 were obtained from Chinese Academy of Sciences (Shanghai, China). All cells were expanded in complete medium (Dulbecco's modified Eagle's medium (DMEM)/F12 medium containing 10% fetal bovine serum (FBS)). Human hsa_circ_0005875 plasmids were synthesized by Genomeditech (Shanghai, China). Cells were transfected with plasmids, or their NC using Lipofectamine 2000 (Invitrogen) based on manufacturer's instructions. The sequences of vector were shown as follows:

hsa_circ_005875-Eco/Bam-F: gtgaccggcgcctacTGTCGAGTTATAGCAGTGC.hsa_circ_005875-Eco/Bam-R: tcgatggaccggtcgCTGGTGCTTGGCAGTTAAA.

### Colony formation assay

ACHN and A498 in the logarithmic growth phase were digested with 0.25% trypsin and beaten into single cells and were plated in 6-well plates and incubated 7days. The cells were cultured until most colonies reached more than 50 cells. Then, the cells were fixed with paraformaldehyde for 30 minutes and stained with 0.1% crystal violet. Finally, cell colonies were counted using Image J and analyzed at the microscope (Tokyo, Japan).

### Transwell migration and Matrigel invasion assays

Cell migration and Matrigel invasion assays were performed by using Transwell chamber (for migration assay) or Transwell which coated Matrigel chamber according to the manufacturer's protocol (BD Bioscience, NJ, USA). The single cell suspensions (0.5 x 10^6^cells/well for migration, 1 × 10^5^/well for invasion) were added to the upper chambers and incubated lasted about 24 h. The migration and invasion cells were stained with 0.1% crystal violet and counted at least three random fields.

### Luciferase reporter assay

293T cells were seeded into 96-well plates at a density of 1×105 cells per well for 24h before co-transfection. The sequences of circ0058792 and their corresponding mutant fragments without hsa-miR-145-5p binding sites were then synthesized and subcloned into luciferase reporter vector GP-miRGLO (GenePharma). After incubate for 48h, firefly and Renilla luciferase activities were analyzed by dual-luciferase reporter assay system (Promega) according to the manufacturer's protocol.

### Statistical analysis

Data are presented as mean ± standard error of the mean (SEM). SPSS 17.0 (SPSS Inc., Chicago, IL, USA) was used for data analysis. The data are presented as mean ± standard error of the mean (SEM) and was normally distributed as examined by Shapiro-Wilk test. An independent-samples *t-*test was also applied to determine correlations between the expression levels of hsa_circ_0005875 and the clinicopathological parameters of the patients with ccRCC. The artworks were created using GraphPad Prism 5.0 (GraphPad Software, La Jolla, CA). Paired-samples *t* test was used for comparing the circRNA expression levels in the tumor versus non-carcinoma tissues. *P* < 0.05 was considered statistically significant.

## Results

### CircRNA expression profiles in ccRCC

**Figure 1:**
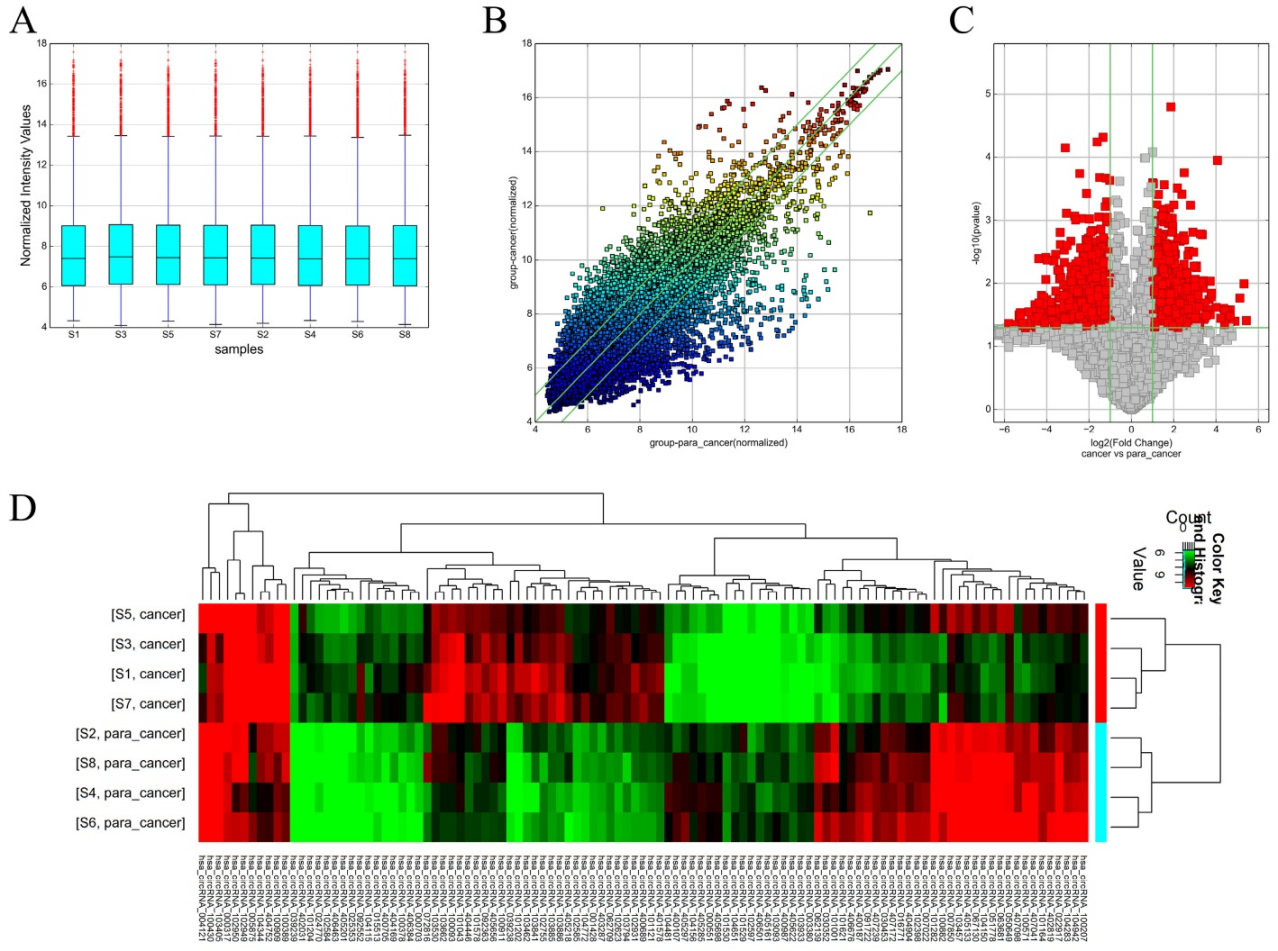
Different expression profiles of circRNAs in ccRCC tissues versus non-carcinoma tissues. (A) The box plot was used for assessing the distributions of circRNA intensities in the tested samples (S1, S3, S5 and S7 are ccRCC tissues while the left are para-carcinoma tissues); the distributions were nearly the same after normalization. (B) The scatter plot revealed microarray data distributions of circRNAs between the ccRCC and the control group. The circRNAs above the top green line and below the bottom line showed more than 2-fold change. (C) Volcano plots revealed significantly dysregulated circRNAs in the ccRCC tissues. The vertical lines represent 2-fold upregulated and downregulated circRNAs, while the horizontal lines indicate that the *P* value is 0.05. Red squares indicate differentially expressed circRNAs in the ccRCC tissues versus the adjacent normal tissues (*P* < 0.05). (D) Unsupervised hierarchical clustering analysis of the circRNAs showing significantly differential expression between the ccRCC tissues and non-carcinoma tissues. Each column represent the expression profile of a sample, while each row corresponds to a circRNA. The color scale varies from red to green; red suggests upregulated circRNAs and downregulated circRNAs are shown in green.

Box plots were used to assess the distributions of the circRNA intensities in the tested samples and the distributions were found to be nearly the same after normalization (**Figure 1A**). The scatter plot revealed microarray data distributions of circRNAs between the ccRCC and the control group (**Figure 1B**). Volcano plots revealed significantly dysregulated circRNAs in the ccRCC tissues and 1988 dysregulated circRNAs in the ccRCC tissues were screened out (**Figure 1C**). Unsupervised hierarchical clustering analysis was then performed for the circRNAs, the heatmap showed significantly differential expression between the ccRCC tissues and para-carcinoma tissues (**Figure 1D**). The top 10 up- and down-regulated circRNAs sorted by fold change (FC) values and *P* values are shown in **Table 2** and **Table 3**, with other detailed information such as chromatin and gene symbols presented.

### Validation of the differentially expressed circRNAs

**Figure 2:**
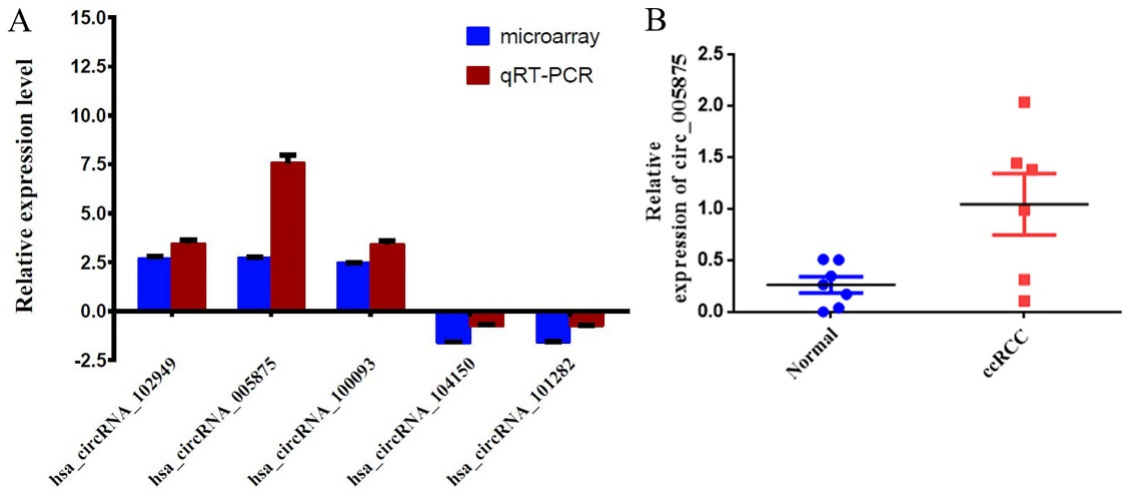
** 2A.** Comparison of circRNA expression between qRT-PCR and microarray analysis results. Four upregulated and two downregulated differentially expressed circRNAs were validated by qRT-PCR. The Y-axis of the columns in the chart represents the log2-transformed median fold changes (C/N) and presented as the mean and standard deviation values.2**B:** The result of circ0005875 on circRNA-sequencing for renal cell carcinoma and control.

The expression of circRNAs were validated by qRT-PCR. Five circRNAs (hsa_circRNA_102949, hsa_circRNA_005875, hsa_circRNA_100093, hsa_circRNA_104150 and hsa_circRNA_101282) were chosen based on the raw intensity and the microRNA binding site. Among them, the expression levels of hsa_circRNA_102949, hsa_circRNA_0005875, hsa_circRNA_100093 were significantly higher in the tumor tissues than in the corresponding non-carcinoma tissues (**Figure 2A**). In contrast, the expression levels of hsa_circRNA_104150 and hsa_circRNA_101282 were significantly decreased in the tumor tissues than in the non-carcinoma tissues (**Figure 2A**). The qRT-PCR results were consistent with the microarray data.

**Figure 3:**
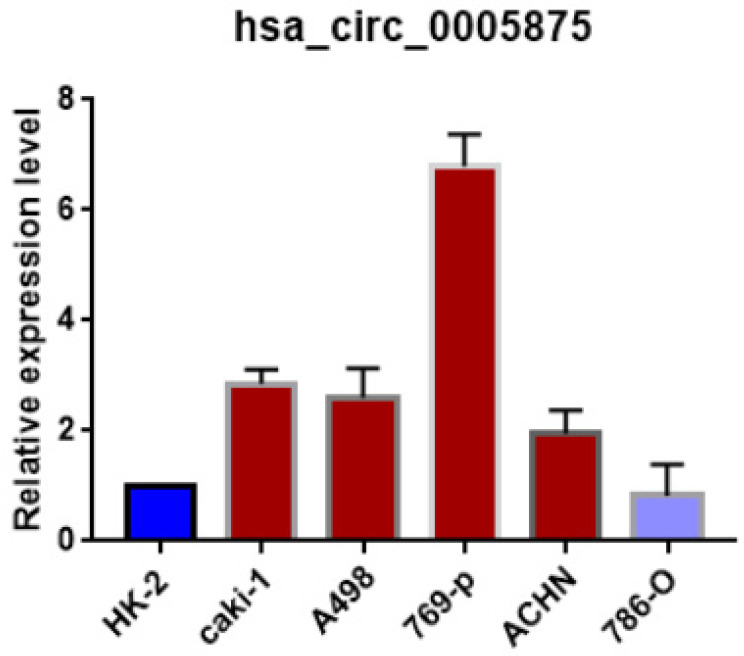
The expression of circ0005875 was determined in 5 ccRCC cell lines and normal HK-2 cell line and values were presented as the mean and standard deviation values.

Moreover, we search the public databases and found that has_circ_0005875 was significantly upregulated in renal cell carcinoma tissues than normal control (accession number: GSE108735; circRNA-sequencing for renal cell carcinoma and control,** Figure 2B**). Meanwhile, the expression of hsa_circRNA_0005875 were analyzed in ccRCC cells. The expression of hsa_circRNA_0005875 were obviously up-regulated in relative ccRCC cell lines except for 786-O cells (**Figure 3**).

### Annotation for circRNA/microRNA interaction

**Figure 4:**
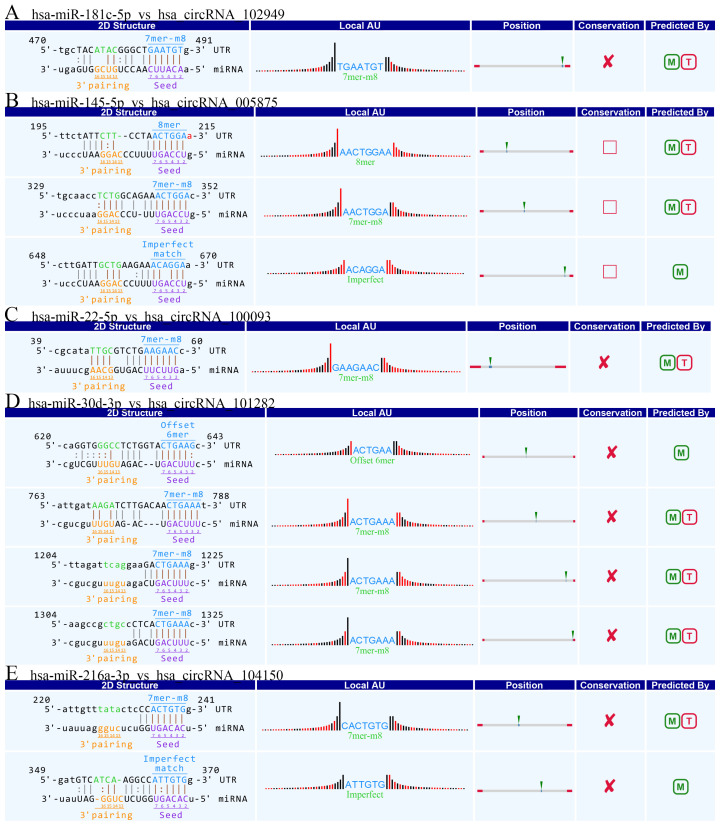
The detailed annotation for circRNA/miRNA interaction. Complementary situations of circRNAs from A to F. (A) hsa_circ_102949. (B) hsa_circ_005875. (C) hsa_circRNA_100093. (D) hsa_circRNA_103349. (E) hsa_circRNA_104150. (F) hsa_circRNA_101282. 7mer-m8: bases from number 2 to 8 match perfectly, and the number 1 base is not A; 8mer: bases from number 2 to 8 match perfectly, and the number 1 base is A; Imperfect match: 2 to7 G:U non-standard matching or mismatch, missing offset; Offset-6mer: bases from number 3-8 match perfectly.

Accumulating evidence suggests that circRNAs play a crucial role in sequestering relevant miRNAs. To investigate the potential functions of circRNAs (Guan M et al., 2016), we explored miRNAs binding MREs (miRNA response elements) with 6 circRNAs (hsa_circRNA_102949, and hsa_circRNA_005875, hsa_circRNA_100093, hsa_circRNA_103349, hsa_circRNA_104150, hsa_circRNA_101282) (**Figure 4**). The MREs with top mirSVR scores for the six confirmed circRNA s are shown in detail.

### Hsa_circ_0005875 as a novel biomarker for ccRCC

We then selected hsa_circ_0005875 for further study. As shown in Table 4, hsa_circ_0005875 expression level was significantly associated with tumor size, pathological TNM stage, histological differentiation, and lymphatic metastasis (**Table 4**). These data suggested that hsa_circ_0005875 might be a potential biomarker of ccRCC.

### Overexpression of hsa_circ_0005875 promoted ccRCC cells proliferation, migration and invasion

**Figure 5:**
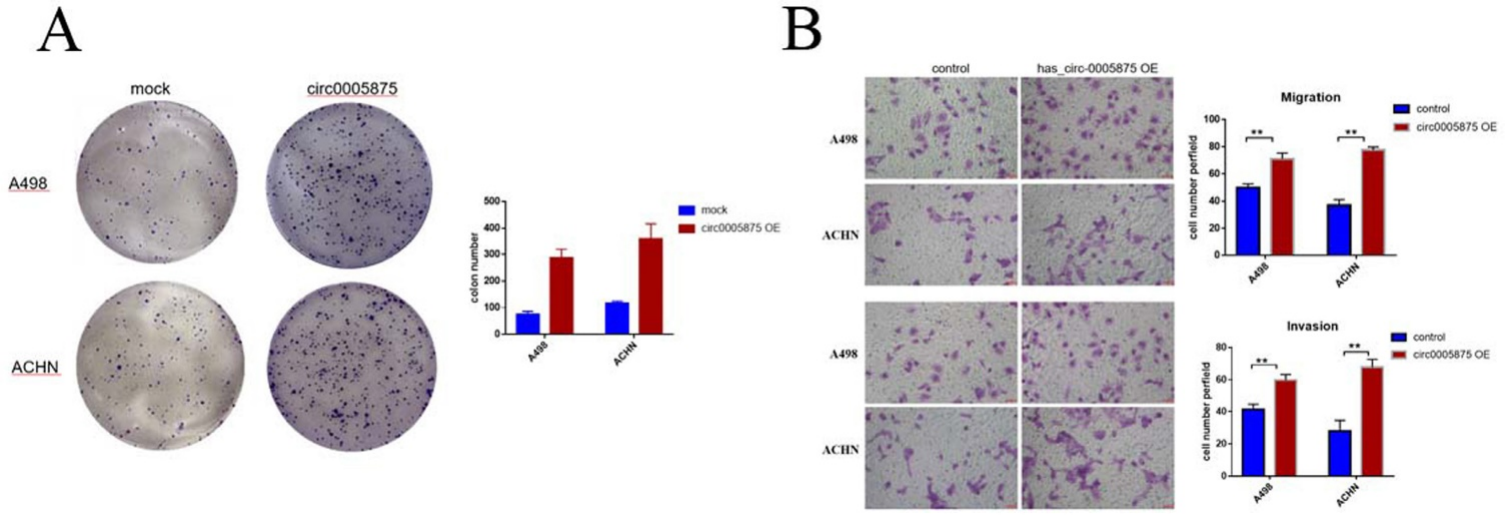
Overexpression of hsa_circ_0005875 promoted ccRCC cells proliferation, migration and invasion. (A) Colony formation assays were executed to detect the proliferation of cells transfected with pcDNA3.1 vector. Data were showed as mean ± SD, *P < 0.05, **P < 0.01. (B) Cell migration and invasion abilities were illustrated that hsa_circ_005875 overexpression promote migration and invasion of A498 and ACHN cells by Transwell and Matrigel-Transwell assays after transfection (magnification, 200; Scale bar, 50 μm)

To explore the biological function of circ_005875 in ccRCC cells, the overexpression vector of hsa_circ_0005875 plasmid was constructed. Colony formation assay demonstrated that upregulation of hsa_circ_0005875 significantly enhanced the proliferation capabilities of ACHN and A498 cells (**Figure 5A**). Transwell and Matrigel-Transwell assay further revealed that hsa_circ_0005875 overexpression induced cell migration and invasion abilities in ccRCC cells (**Figure 5B**).

### Prediction and annotation of hsa_circ_0005875-targeted miRNA-mRNA network

**Figure 6:**
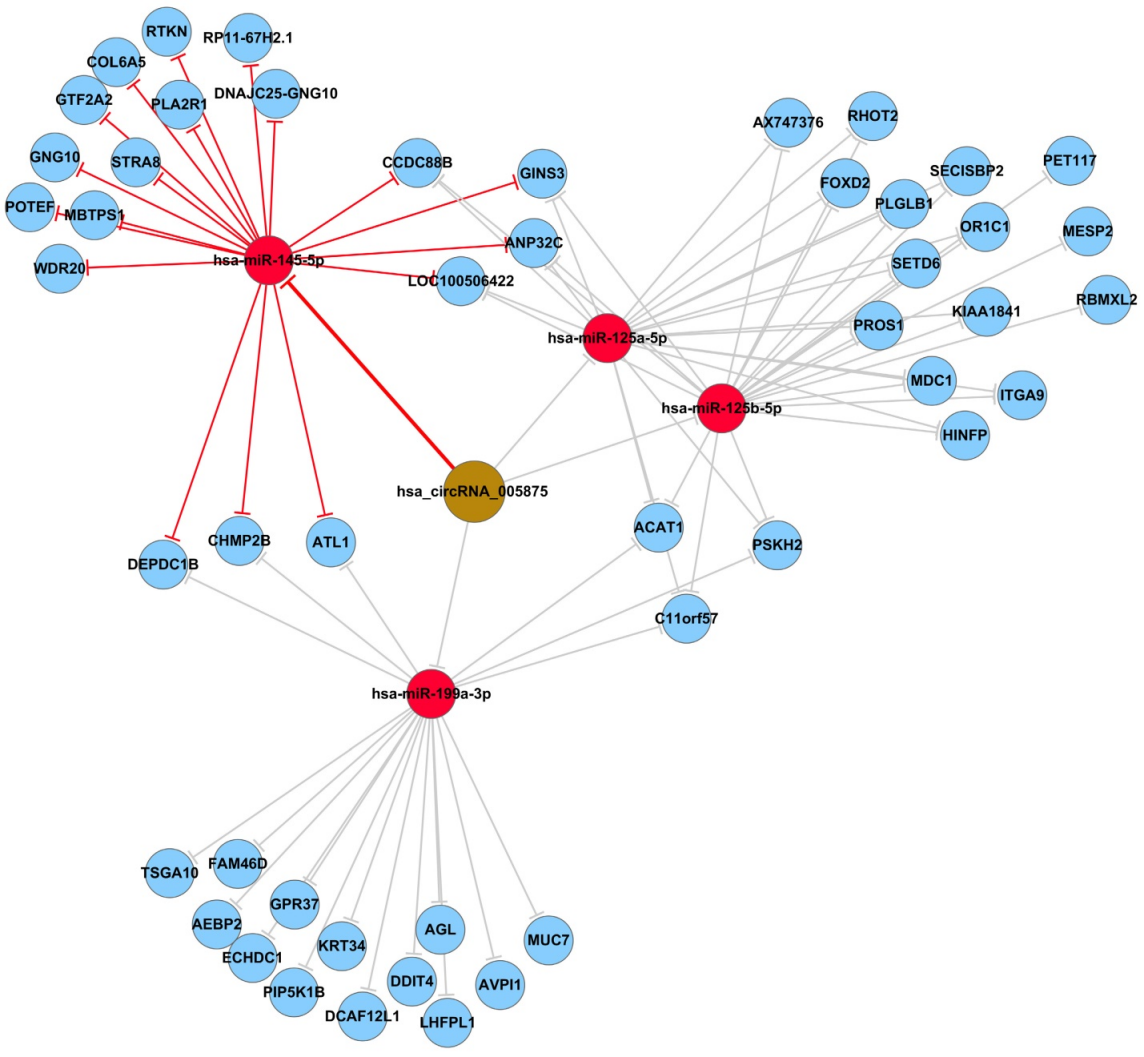
The predicted hsa_circ_005875-targeted circRNA-miRNA-mRNA gene network. All the miRNA-binding sites predicted based on mirSVR scores, and targeted miRNAs and mRNAs predicted using TargetScan and miRanda. As shown in this network, hsa-miR-125a-5p, hsa-miR-125b-5p, hsa-hsa-miR-145-5p, and hsa-miR-199a-3p were the highest differences in expression.

To identify the target miRNAs for hsa_circ_0005875, cytoscape analysis of the circRNA-miRNA-mRNA interaction network of hsa_circ_0005875 indicated that hsa-miR-125a-5p, hsa-miR-125b-5p, hsa-hsa-miR-145-5p, and hsa-miR-199a-3p as the most closely related miRNAs with respective potential target mRNAs (**Figure 6**).

### Hsa_circ_0005875 functions as a sponge for hsa-miR-145-5p

**Figure 7:**
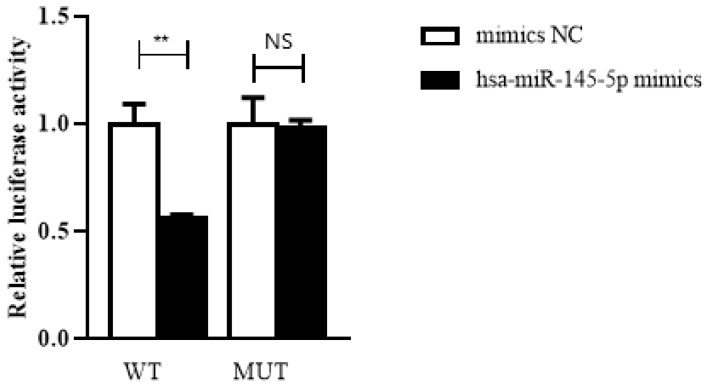
Hsa_circ_0005875 functions as a sponge for hsa-hsa-miR-145-5p. The relative luciferase activities were analyzed in HEK293T cells co-transfected with miR-15a-5p mimics, miR-mimics-NC and WT or Mut luciferase reporter vectors. **P < 0.01, N.S, nonsignificant

To confirm the prediction, dual-luciferase reporter assay was performed in HEK293T cells. The luciferase reporters were cotransfected with hsa-miR-145-5p mimics or NC mimics into HEK293T cells. We observed that the luciferase activity of WT reporters cotransfeced with hsa-miR-145-5p mimics was remarkably decreased whereas the luciferase activity of MUT ones showed no significantly changes (**Figure 7**).

### GO analysis of circRNA gene symbols and pathway analysis

**Figure 8:**
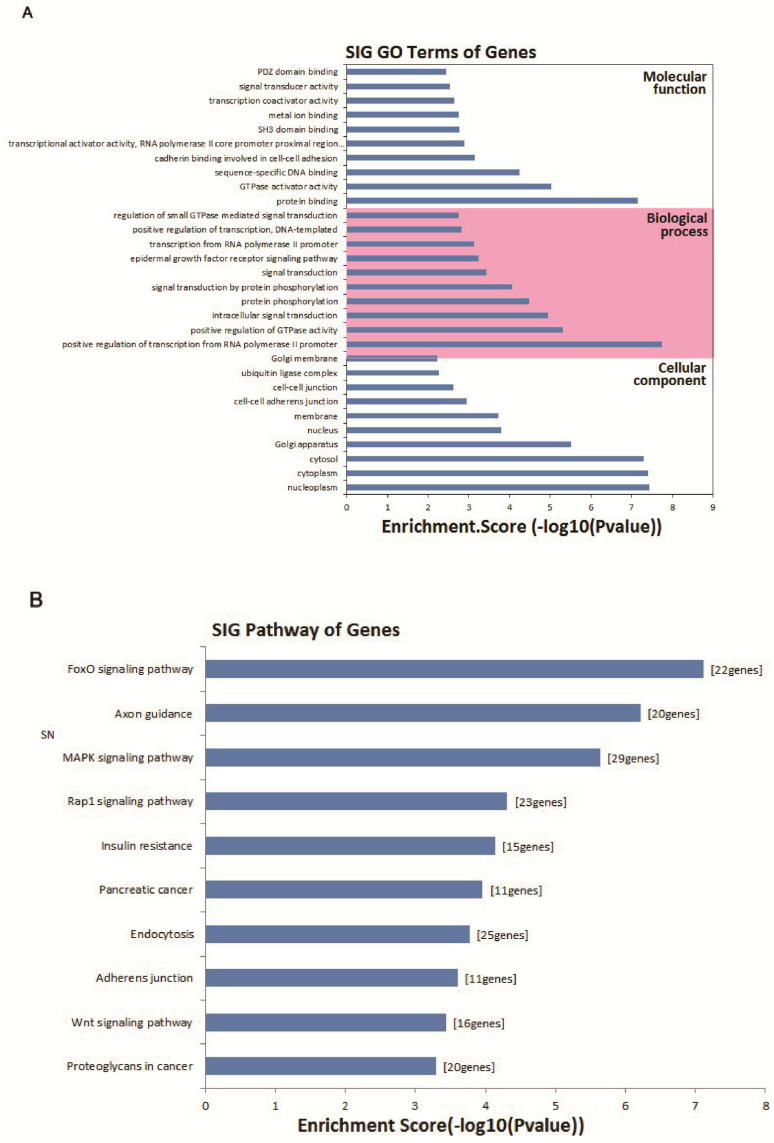
Bioinformatics analysis of has_circ_0005875-targeted circRNA-miRNA-mRNA networks. (A) GO analysis. (B) Target mRNAs of the networks were functionally annotated using KOBAS and then KEGG pathway analysis was performed. Top 10 significantly enriched pathway terms of the networks are shown.

To evaluate the attributes of hsa_circ_0005875 in terms of molecular functions, biological process, cellular components and pathways, we conducted GO analysis and KEGG pathway analysis for circRNA gene symbols to determine the potential functions of hsa_circ_0005875 based on the results from TargetScan and miRanda. We found that the most significantly enriched GO term in the biological process was positive regulation of transcription by the RNA polymerase II promoter; the most significantly enriched GO term in the cellular component was nucleoplasm; and the most significantly enriched GO term in the molecular function was protein binding (**Figure 8A**). Pathway analysis indicated that 10 pathways might be involved in the progression of ccRCC (**Figure 8B**).

## Discussion

In this study, we identified a large number of circular RNAs in ccRCC tissues by circRNA microarray. In total, 1988 circRNAs (FC ≥ 2.0, *P* value < 0.05), including 1033 upregulated and 955 downregulated ones, were found to be significantly dysregulated in the ccRCC tissues. The data suggested that circRNAs play a crucial role in the pathogenesis of ccRCC.

The relationship between hsa_circ_0005875 and clinical parameters indicated that hsa_circ_0005875 might be a potential biomarker of ccRCC. Functional experiment confirmed that overexpression of hsa_circ_0005875 could promote ccRCC cells proliferation, mobility and invasion *in vitro*. The bioinformatics analysis showed that hsa_circ_0005875 may function as miRNA sponges and was predicted to be associated with the biological process of the epidermal growth factor receptor signaling pathway, involving in cancer invasion and metastasis. The genomic length of hsa_circ_0005875 is 12846 bp, and the spliced mature sequence length is 873 bp. It is located at chr3:132337477-132350323. It has been proven to be related with end-stage kidney disease 14, but the investigation of hsa_circ_0005875 in tumors is still lacking. In recent years, more and more circRNAs have been found to play important roles in the development of tumors and can therefore function as potential tumor markers of those tumors. For example, circNR1P1 was reported to be involved in regulating the growth and metastasis of gastric cancer by regulating the expression of cyclinD1, CDK6, MMP-2, and MMP-9 15.

CircHIPK3 is also highly expressed in colorectal cancer and has been researched to be closely related with patient age, tumor size, and TNM staging 16. Similarly, down-regulation of circ0067934 significantly inhibits the proliferation, metastasis, and cell cycle of esophageal squamous cell carcinoma; therefore, circGSK3β can function as the tumor marker of esophageal squamous cell carcinoma 17. Furthermore, circTCF25 can promote the proliferation and metastasis of bladder cancer by downregulating the expression of miR-103a-3p and miR-107, and therefore can serve as a new therapeutic target and tumor marker of bladder cancer 18. Similarly, based on our results, we speculate that hsa_circ_0005875 plays a regulatory role in the biological processes of ccRCC and therefore believe that it might be a potential biomarker of ccRCC. The further experiment on the function and mechanism to verify this speculation will be conducted in the near future.

One of the well-recognized functions of circRNAs is as sponging miRNAs in circRNA-miRNA-mRNA regulatory axes. In this study, using the TargetScan/miRanda software, hsa_circ_0005875 was found to potentially interact with miR-125a-5p, miR-125b-5p, hsa-miR-145-5p, and miR-199a-3p. Among them, hsa-miR-145-5p can function as a tumor-suppressor miRNA and has been proven to be involved in regulating the development of multiple tumors 19. Dual-luciferase reporter assay showed that hsa_circ_0005875 may function as miR-145-5p sponge. According to previous studies, the downstream target gene of hsa-miR-145-5p is ZEB2 20. ZEB2 is a member of the ZEB transcription factor family and plays a role in the development and progression of malignant tumors by regulating the EMT process 21. ZEB inhibits the expression of E-cadherin by binding independently to the [CACCT (G)] sequences of the E-cadherin promoter, which results in epithelial-mesenchymal transformation, thus enhancing cell metastasis and invasion 22. ZEB2 has been shown to enhance the invasion and metastasis of a variety of malignant tumors including colorectal cancer 23, gastric cancer 24, breast cancer 25, bladder cancer 26, and nasopharyngeal carcinoma 27. Recent studies have also shown that ZEB2 is highly expressed in ccRCCs and that its expression level is closely related to progression-free and overall survival rate of renal cell carcinoma 28. ZEB2 has also been reported as an independent prognostic indicator of ccRCC, and downregulation of ZEB2 significantly inhibits the invasion and migration of ccRCC 29. In the present study, we predicted that the hsa_circ_0005875/hsa-miR-145-5p/ZEB2 pathway plays a very important role in ccRCC; however, the mechanism underlying this role needs further investigation to be confirmed.

To the best of our knowledge, this is one of few studies to reveal that numerous circRNAs are dysregulated in ccRCC tissues. We also found that hsa_circ_0005875 interacts with hsa-miR-145-5p. The hsa_circ_0005875/hsa-miR-145-5p/ZEB2 pathway will be further verified in our future studies. In addition, the function of hsa_circ_0005875 in ccRCC also needs to be further explored in both in vitro and in vivo studies. In the future, hsa_circ_0005875-stably downregulated cell lines and the detailed molecular mechanisms by which hsa_circ_0005875 contributes to ccRCC proliferation, invasion, and metastasis need to be further investigated. Furthermore, whether hsa_circ_0005875 can be utilized as a novel biomarker for ccRCC needs to be further verified by larger clinical samples.

However, our study has some shortcomings that should be acknowledged. Firstly, we collected samples only grouped into carcinoma and adjacent tissue, further study will divide the samples in a more detailed sample on early phase and late phase. Secondly, the expression of circRNAs are all detected in ccRCC tissues, further study will detect them in blood and urine on the basis of a larger ccRCC tissues sample so as to find a suitable biomarker. Last but not the least, we understand that functional experiment may better sustain our hypotheses. However, in the present study, we mainly focused on some candidate circRNAs including hsa_circ_0005875 which can act as potential biological marker, and we think that available experiment data may not be optimal, unfortunately, results are unavailable at this point. It will be of great interest to initiate some research on the function and expression of ccRCC in our further study.

## Supplementary Material

Supplementary table.

## Figures and Tables

**Table 1:** Clinical characteristics of the patients for microarray analysis.

Patients	Sex	Age	Histological Differentiation	Clinical stage	Lymphatic Metastasis
1	female	51	moderate	I	No
2	female	65	poor	III	Yes
3	male	49	moderate	I	No
4	male	70	poor	III	Yes

**Table 2:** Top 10 upregulated circRNAs in the ccRCC tissues screened by fold changes (FC) and *P* value. CircRNA ID was based on circBase (http://www.circbase.org/).

circRNA	P-value	FDR	FC (abs)	chrom	strand	GeneSymbol
hsa_circRNA_401696	0.039	0.239	43.021	chr17	-	ANKFY1
hsa_circRNA_101341	0.010	0.233	39.957	chr14	-	EGLN3
hsa_circRNA_405198	0.017	0.234	34.885	chr14	-	EGLN3
hsa_circRNA_092390	0.039	0.239	29.713	chr11	+	PPP6R3
hsa_circRNA_102950	0.040	0.239	25.425	chr2	+	AGAP1
hsa_circRNA_001873	0.033	0.238	25.394	chr9	-	SYK
hsa_circRNA_406752	0.026	0.235	25.360	chr6	+	PRRC2A
hsa_circRNA_402565	0.027	0.235	23.256	chr20	-	EDEM2
hsa_circRNA_100703	0.031	0.237	22.138	chr10	-	CHST15
hsa_circRNA_102949	0.035	0.239	21.420	chr2	+	AGAP1
hsa_circRNA_006562	0.027	0.235	21.344	chr7	-	LOC493754

**Table 3:** Top 10 downregulated circRNAs in the ccRCC tissues screened by fold changes (FC) and *P* value. CircRNA ID was based on circBase (http://www.circbase.org/).

circRNA	*P*-value	FDR	FC (abs)	chrom	strand	GeneSymbol
hsa_circRNA_062139	0.002	0.233	10.338	chr22	+	TPTEP1
hsa_circRNA_067130	0.002	0.233	10.252	chr3	-	ZXDC
hsa_circRNA_063681	0.003	0.233	11.963	chr22	-	TTLL1
hsa_circRNA_101001	0.004	0.233	16.491	chr12	-	SCNN1A
hsa_circRNA_004121	0.004	0.233	20.783	chr2	-	RFX8
hsa_circRNA_051778	0.004	0.233	15.788	chr19	-	BCAT2
hsa_circRNA_030352	0.004	0.233	12.689	chr13	+	TPTE2P3
hsa_circRNA_103561	0.006	0.233	10.496	chr3	+	SENP5
hsa_circRNA_104150	0.006	0.233	11.133	chr6	-	ME1
hsa_circRNA_001257	0.008	0.233	10.086	chr22	-	PLXNB2
hsa_circRNA_000480	0.008	0.233	10.511	chr13	+	ZC3H13

**Table 4:** Correlation between hsa_circ_0005875 expression and clinical parameters in patients with ccRCC.

Characteristics	No. Patients (%)	mean±SD	*T* value	*P* value
Sex				
Female	28(46.7)	9.34±2.06	0.492	0.624
Male	32(53.3)	9.68±3.19		
Age (years old)				
<60	34 (%56.67)	8.99±2.44	1.740	0.087
≥60	26 (%43.33)	10.17±2.77		
Tumor Size				
≥5	24(40.00)	11.55±2.07	7.963	0.000
<5	36(60.00)	7.78±1.66		
nuclear grade				
G1-2	37(61.67)	8.826±2.14	2.540	
G3-4	23 (%38.33)	10.68±3.02		0.016
clinical Stage				
Ⅰ+Ⅱ	38 (%63.33)	8.56 ±2.03	3.646	0.001
Ⅲ+Ⅳ	22 (%36.67)	11.02± 2.81		
Lymphatic Metastasis				
Yes	17 (%28.33)	11.45±2.74	4.052	0.000
No	43 (%71.67)	8.73±2.17		
