# Supplementary material for: Circular RNA microarray expression profile and potential function of circ0005875 in clear cell renal cell carcinoma
Source: J Cancer. 2020 Oct 18;11(24):7146–56. doi: 10.7150/jca.48770 (PMC7646169; doi:10.7150/jca.48770)
Supplement: Supplementary file 1 — Supplementary table. [file jcav11p7146s1.pdf]

supplementary table 1:

NO.	gender	age	Symptom/Physical signs	Left/right kidney	size	nuclear grade	TNM (clinical)stage	Lymphatic Metastasis
1	male	73	None	left	4.2cm×4.3cm	G2	$T_{1b}N_0M_0$(I)	NO
2	female	74	Hematuria/abdominal palpable mass	right	12cm×8.6cm	G4	$T_{3a}N_1M_0$(III)	YES
3	male	67	None	right	7.5cm×5.2cm	G3	$T_2N_1M_0$(III)	YES
4	male	61	None	left	3.6cm×2.9cm	G1-2	$T_{1a}N_0M_0$(I)	NO
5	male	64	None	left	3.0cm×2.8cm	G2	$T_{1a}N_0M_0$(I)	NO
6	male	62	None	right	4.9cm×4.5cm	G3	$T_{1a}N_0M_0$(I)	NO
7	male	73	None	right	2.7cm×2.4cm	G2	$T_{1a}N_0M_0$(I)	NO
8	male	51	Fever	left	10cm×7.2cm	G3	$T_{3a}N_1M_0$(III)	YES
9	male	70	None	left	1.6cm×1.6cm	G2	$T_{1a}N_0M_0$(I)	NO
10	male	73	None	right	4.0cm×3.5cm	G2	$T_{1a}N_0M_0$(I)	NO
11	male	63	None	left	5.6cm×5.0cm	G3	$T_{1b}N_0M_0$(I)	NO
12	male	70	Hematuria&Fever	right	9.5cm×9.0cm	G3-4	$T_2N_0M_1$(IV)	NO
13	male	78	Hematuria/abdominal palpable mass	left	4.5cm×4.6cm	G3	$T_{1b}N_0M_0$(I)	NO
14	male	47	None	right	3.6cm×3.1cm	G2	$T_{1a}N_0M_0$(I)	NO
15	male	73	None	right	4 cm×3.6cm	G2	$T_{1a}N_1M_0$(III)	YES
16	female	52	None	right	3cm×3cm	G2	$T_{1a}N_0M_0$(I)	NO
17	male	57	Macroscopic hematuria	left	9.5cm×8.6cm	G2	$T_2N_0M_1$(IV)	NO
18	female	49	None	left	2.6cm×2.5cm	G1	$T_{1a}N_0M_0$(I)	NO
19	male	48	None	right	3.2cm×2.7c	G1-2	$T_{1a}N_0M_0$(I)	NO

					m			
20	female	46	None	right	3.7cm×3.1cm	G1	$T_{1a}N_1M_0$(III)	YES
21	male	52	None	left	2.8cm×2.2cm	G1-2	$T_{1a}N_0M_0$(I)	NO
22	male	63	Back pain	left	6.7cm×5.1cm	G2	$T_{1b}N_1M_0$(III)	YES
23	male	63	Hematuria/abdominal palpable mass	right	9.6cm×7.5cm	G3	$T_2N_1M_1$(IV)	YES
24	female	59	None	left	2.1cm×1.8cm	G1-2	$T_{1a}N_0M_0$(I)	NO
25	male	73	None	right	5.9cm×5.7cm	G3	$T_{1a}N_0M_0$(I)	NO
26	male	45	Hematuria	right	4.9cm×4.7cm	G1	$T_{1b}N_1M_0$(III)	YES
27	male	65	Back pain	right	9.5cm×7cm	G3	$T_2N_1M_0$(III)	YES
28	female	47	None	right	5.1cm×5.7cm	G3	$T_{1a}N_0M_0$(I)	NO
29	female	74	Macroscopic hematuria	left	11cm×10cm	G2	$T_2N_1M_0$(III)	YES
30	female	52	None	right	3.0cm×2.7cm	G1	$T_{1a}N_0M_0$(I)	NO
31	female	72	None	right	8 cm×7.5cm	G3	$T_2N_1M_0$(III)	YES
32	male	58	None	right	6.5cm×5.9cm	G3	$T_{1b}N_0M_0$(I)	NO
33	female	70	Back pain&Fever	right	7 cm×6.5cm	G4	$T_1N_0M_1$(IV)	NO
34	male	59	None	left	6.3cm×5.1cm	G1-2	$T_{1a}N_0M_0$(I)	NO
35	female	80	None	right	2.5cm×2.0cm	G1-2	$T_{1a}N_0M_0$(I)	YES
36	male	42	None	right	4.7cm×4.5cm	G3-4	$T_{1a}N_0M_0$(I)	NO
37	female	60	None	right	1.5cm×1.5cm	G1	$T_{1a}N_0M_0$(I)	NO
38	female	38	None	left	6.5cm×5.5cm	G1-2	$T_{1b}N_0M_1$(IV)	NO
39	female	65	None	right	1.8cm×1.6cm	G1-2	$T_{1a}N_0M_0$(I)	NO
40	male	55	None	left	2.7cm×2.3cm	G1-2	$T_{1a}N_0M_0$(I)	NO

41	female	69	Hematuria/back pain	right	8.5cm×8.2cm	G1-2	$T_{2a}N_1M_0$(III)	YES
42	female	64	None	left	6.2cm×5cm	G2	$T_{1b}N_1M_0$(III)	YES
43	female	57	None	right	8.6cm×8cm	G3-4	$T_{2a}N_1M_0$(III)	YES
44	male	76	None	left	3.1cm×2.5cm	G1-2	$T_{1a}N_0M_0$(I)	NO
45	female	58	None	left	1.2cm×1.0cm	G1	$T_{1a}N_0M_0$(I)	NO
46	female	63	None	left	2.6cm×2.6cm	G3	$T_{1a}N_0M_0$(I)	NO
47	female	74	None	right	4.6cm×4.5cm	G3	$T_{1b}N_0M_0$(I)	NO
48	male	52	None	left	3.2cm×2.7cm	G2	$T_{1a}N_0M_0$(I)	NO
49	female	73	None	right	4.7cm×4.1cm	G3	$T_{1a}N_0M_0$(I)	NO
50	female	61	None	right	2.8cm×2.2cm	G1-2	$T_{1a}N_0M_0$(I)	NO
51	female	78	None	left	1.6cm×1.5cm	G1	$T_{1a}N_0M_0$(I)	NO
52	female	47	Back pain	right	8.9cm×8.5cm	G3	$T_{2a}N_1M_0$(III)	YES
53	female	53	None	left	1.8cm×1.6cm	G1	$T_{1a}N_0M_0$(I)	NO
54	male	57	None	right	2.5cm×1.8cm	G1-2	$T_{1a}N_0M_0$(I)	NO
55	male	42	None	left	2.6cm×2.0cm	G1-2	$T_{1a}N_0M_0$(I)	NO
56	male	48	Cough	right	7.5cm×6.8cm	G3	$T_{2a}N_0M_1$(IV)	NO
57	male	56	Back pain	left	8.6cm×8.3cm	G3-4	$T_{2a}N_0M_0$(II)	NO
58	female	78	None	right	2.1cm×1.8cm	G1	$T_{1a}N_0M_0$(I)	NO
59	female	64	None	left	3.7cm×2.8cm	G2	$T_{1a}N_0M_0$(I)	NO
60	female	64	Macroscopic hematuria	right	4.8cm×4.7cm	G3-4	$T_{1b}N_1M_0$(III)	YES
61	female	51	None	right	2.8cm×2.5cm	G2	$T_{1a}N_0M_0$(I)	NO
62	fema	65	None	left	4.5cm×4.8c	G3-4	$T_{1b}N_1M_0$(	YES

	le				m		III)	
63	male	49	None	right	2.1cm×2.0cm	G1-2	$T_{1a}N_0M_0$(I)	NO
64	male	70	None	right	5.8cm×4.8cm	G3	$T_{1b}N_1M_0$(III)	YES

*By AJCC 2017 8th TNM staging edition

The information of last four samples is for microarray anlysis.
